# Why Are Digital Health Care Systems Still Poorly Designed, and Why Is Health Care Practice Not Asking for More? Three Paths Toward a Sustainable Digital Work Environment

**DOI:** 10.2196/26694

**Published:** 2021-06-22

**Authors:** Johanna Persson, Christofer Rydenfält

**Affiliations:** 1 Department of Design Sciences Lund University Lund Sweden

**Keywords:** digital systems, electronic health records, digital work environment, ergonomics, usability, human-centered design

## Abstract

Knowledge of how to design digital systems that are ergonomically sound, high in usability, and optimized for the user, context, and task has existed for some time. Despite this, there are still too many examples of new digital health care systems that are poorly designed and that could negatively affect both the work environment of health care staff and patient safety. This could be because of a gap between the theoretical knowledge of design and ergonomics and the practical implementation of this knowledge in procuring and developing digital health care systems. Furthermore, discussions of digitalization are often at a general level and risk neglecting the nature of direct interaction with the digital system. This is problematic since it is at this detailed level that work environment and patient safety issues materialize in practice. In this paper, we illustrate such issues with two scenarios concerned with contemporary electronic health care records, based on field studies in two health care settings. We argue that current methods and tools for designing and evaluating digital systems in health care must cater both to the holistic level and to the details of interaction and ergonomics. It must also be acknowledged that health care professionals are neither designers nor engineers, so expectations of them during the development of digital systems must be realistic. We suggest three paths toward a more sustainable digital work environment in health care: (1) better *tools for evaluating the digital work environment in the field*; (2) *generic formulations of qualitative requirements* related to usability and for adaptation to the user, context, and task, to be used in procurement; and (3) the introduction of *digital ergonomics* as an embracing concept capturing several of the ergonomic challenges (including physical, cognitive, and organizational aspects) involved in implementing and using digital systems.

## Introduction

There is no doubt that the digital transformation of health care is changing how health care is delivered [1,2]. Besides aspects of efficiency, safety, and patient empowerment, digitalization also affects the work environment of health care staff. New digital systems can both facilitate and constrain work, depending on their design and how well they support organizational goals and objectives [3-5]. Furthermore, technology is never neutral. Verbeek [6] notes the following:

When technologies are used, they always help to shape the context in which they fulfill their function. They help to shape human actions and perceptions, and create new practices and ways of living.

Researchers have studied the impact of digital systems on the work environment since the early eighties [7-11]. Since then, diverse digital systems have emerged, ranging from personal computers for rudimentary office tasks [12] to more sophisticated systems, such as systems for computer-supported collaborative work [13,14], or mobile devices and integrated software and hardware [15,16].

In parallel with the introduction of digital systems in the workplace, the field of human-computer interaction (HCI) has developed, and the usability (ie, the efficient, effective, and satisfactory use of a system with respect to the specified user, goal, and use context [17]) of digital systems in health care has become a broadly used concept [18-26]. The idea of involving users in software development to ensure the creation of systems with relevant functionality and high usability had already been introduced in the eighties [27]. Along with this, HCI design principles emerged, intended to match the system to the user’s cognitive abilities, such as limitations of memory, the perceptual system, and mental models [28-30]. A user-centered design process and HCI design principles still constitute the foundation of both HCI education and practice. Nowadays, there is even a standardized process for developing new digital systems intended to ensure that human perspectives are considered [31]. Furthermore, many engineers and developers with HCI knowledge are graduating from higher education institutions worldwide [32-34]. Together, this implies the existence of the technical proficiency and maturity required to develop digital systems with relevant functionality and good usability.

Despite this, basic usability-related problems continue to emerge in health care practice [5,35-37]. Issues related to efficiency, safety, and work environment follow in the tracks of the digital transformation of health care. The problems have even been referred to as “the other health care crisis” (the first one being the COVID-19 pandemic) [38], and links to job frustration and burnout have been investigated [39]. Solutions are hence asked for, and the search for explanations is ongoing [40-42]. We ask ourselves why this is still so when the conditions for developing usable and efficient digital systems, based on HCI design principles and user-centered design methods, have never been better.

In this paper, we identify new ways to overcome obstacles related to poorly designed digital health care systems. Specifically, we highlight the potential effects on the work environment of violations of HCI design principles and of deficient knowledge of the user, use context, and task. This is illustrated through the analysis of two scenarios, providing insights into how these shortcomings materialize in health care practice. Based on insights from these scenarios, we argue for three paths forward that we believe could lead to a more ergonomically sound digitalization of health care.

## Effects of Poorly Designed Digital Systems on Health Care Professionals’ Work Environment

### Two Scenarios

In the following section, we first present two scenarios concerning the use of contemporary electronic health care records (EHRs). EHRs are used as a means to demonstrate our viewpoints, as they are used by basically everyone in the health care sector, whether you are in home care, in primary care, or in specialized care, and whether you are a doctor, a nurse, a lab assistant, or an administrator. They are, furthermore, frequently used throughout the day. We then outline four insights from the scenarios built on basic HCI theory, which illustrate how digital systems affect health care professionals’ work environment.

The two scenarios are synthesized from real situations witnessed in data from in vivo observations of Swedish home health care and primary care performed in 2014 and 2018 as part of two research projects. One project aimed at studying digital systems used by practitioners in home health care. The other one studied the implementation of a new EHR for primary care and included both an expert evaluation of the usability of the system as well as in vivo observations of the system at primary care centers.

#### Scenario 1: Home Health Care

On Monday morning, Anna, a 47-year-old registered home health care nurse, logs in to her computer and opens the EHR to get an update on what has happened with her patients since she last saw them on Thursday the week before. She starts by looking through the messages sent by the staff working during the weekend. There are 51 new messages in the message box. With the introduction of the new EHR system, the number of messages has multiplied due to a new way of sharing information in which messages are sent to work groups instead of individuals. Many of the messages are obsolete because they were already addressed during the weekend, while others are not relevant to Anna’s work. She understands that she must read 20%-25% of the messages more carefully and that the only way to find them is to go through the list one message at a time and delete the irrelevant ones.

Anna needs to check what has happened to one of her patients during the weekend, so she opens the health record for that patient. The information is sorted chronologically with the oldest post first, and since the patient has had home health care for 3 years, the health record is long. To find the most recent post from the past weekend, Anna must scroll through several pages of records. To avoid this time-consuming activity, which entails a lot of clicking, Anna has learned that she can access the information in reverse chronological order by creating a PDF file for printing. By using this workaround, she can access the information she wants about the patient more quickly.

**Figure 1:**
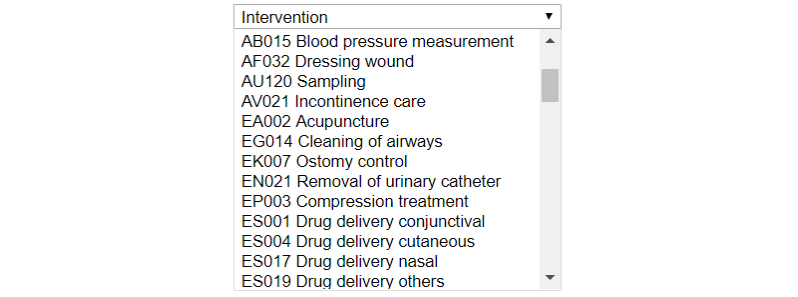
An illustration of a drop-down menu with interventions sorted by code and not by name. The user must scroll through the list to find the desired intervention.

In the afternoon, Anna returns to the office from a patient visit and opens the EHR to document the visit. This procedure is by far the one most often performed in the system. The process is cumbersome. Besides the actual writing, it takes Anna 19 clicks to find the patient, open the patient’s health record, locate the correct place to insert information, and then verify and save the information. To make things even more annoying, the system takes more than a second to respond to each of Anna’s clicks, with the consequence that she makes an extra click or two just to be sure. As part of the documentation process, Anna must also classify the intervention performed at the patient’s home. Each intervention has a name, for example, “Ostomy control,” and a code, for example, “EK007,” selected from a drop-down menu with a long list of all available interventions (Figure 1). However, the list of interventions is not sorted by intervention name but by intervention code, which is not in line with how Anna stores the information in her head. Anna starts reading every line in the long list, and when she comes to the end of the page, she scrolls down. Finally, she finds what she is looking for: “EK007 Ostomy control.” Anna marks the list entry using the cursor and clicks “Save.”

#### Scenario 2: Primary Care

**Figure 2:**
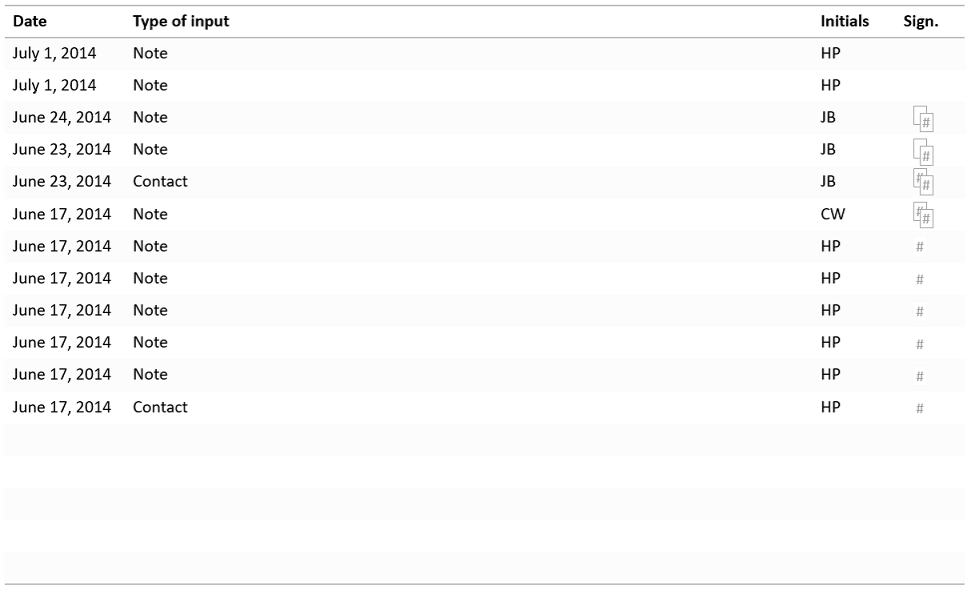
An illustration of how one view of the health record for one patient can look in the system. There is one input line per date, and all inputs here are either notes or information about the patient contact made. The “Initials” column shows who made the input, and the “Sign.” column shows the signing status: an empty space indicates that all signing has been completed; a single hashtag indicates that signing needs to be done; the overlapping hashtags (one or two) indicate that a post was signed but then changed and needs to be looked at and signed again.

**Figure 3:**
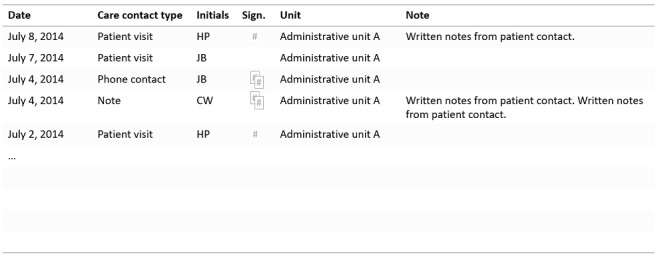
A different electronic health care record layout. This view presents information organized differently from the view shown in Figure 2; for example, the signing icons now appear in the center of the table and not to the right.

General practitioner Helen is finishing her shift at the primary care center. Before she can go home, she has a long list of EHR posts to verify and sign. She has postponed this activity for too long since it is so cumbersome to perform. There are several ways this signing can be done, but she has not found a way that suits the workflow and that she can easily remember, so she is unsure whether she is doing it correctly. She needs to stay focused when jumping between the view in which she keeps track of which posts are still unsigned and the view in which the actual information to be signed off is presented. Furthermore, the feedback on each post is difficult to grasp. A post with a certain hashtag symbol has been signed and needs no further attention. Two smaller, overlapping hashtag symbols indicate a post that was signed but has been changed and thus must be viewed and signed again. A post that is unsigned has no symbol at all and is thus not highlighted in any way (Figure 2). The symbols are quite small and appear in different locations in different views in the system (Figure 2, Figure 3), so Helen must search to find them when switching between views.

Helen is interrupted in her work by a medical laboratory assistant. He wants Helen to change the lab test order she placed for a patient. The lab test that Helen selected from the list in the EHR system cannot be performed at this primary care center. It is not that she did not know this, but that the list from which lab tests are chosen is long and the names are often similar, so she sometimes clicks on the wrong one. Helen is not the only physician making this mistake, and to prevent this from happening, the medical laboratory has printed a separate list on paper showing only the available lab tests. Helen is annoyed that she has to interrupt her work to change the lab test order. She opens the list of lab tests on the computer, verifies that she has now chosen the correct one by double-checking against the printed list, and then finishes the signing before going home 25 minutes later than planned.

### Insights From the Scenarios

The scenarios have been synthesized from situations witnessed in health care practice to illustrate what working in a state-of-the-art EHR can be like. Several insights (Textbox 1) can be derived from these scenarios in order to understand how details in the design of and interaction with a digital system affect the work environment. The analysis of the scenarios leading to these insights is based on knowledge about how user-centered design processes and HCI design principles are aimed to guide the design of systems to avoid exactly the kind of situations demonstrated in the scenarios.

**Overview of insights derived from studying the usage of digital systems in contemporary health care practice.**

- Insight 1: The digital system forces the user into inefficient workflows
- Insight 2: HCI design principles—independent of user, task, and context—are not adopted
- Insight 3: Workarounds become permanent solutions
- Insight 4: Different domains of ergonomics interact in creating the digital work environment

#### Insight 1: The Digital System Forces the User Into Inefficient Workflows

How different functions in the digital system are designed forces the user to adapt how tasks are performed. When the new EHR was introduced in the home health care organization (scenario 1), the communication routine changed. The design of the new system increased the number of messages that the user had to process each day. Similarly, the general practitioner at the primary care center (scenario 2) postponed the signing task since the system hindered rather than supported her in this process by not providing an intuitive workflow or a consistent design. This resulted in an inefficient procedure, causing both frustration and physical strain due to excessive clicking. Demands for more extensive signing in the primary care EHR (scenario 2) were deliberately introduced as a new work routine when implementing the new EHR in order to increase patient safety. Hence, the extra effort required to perform this task may be justified. However, how it was implemented in the system, with a confusing workflow, poor feedback, and inconsistency, could have been avoided by applying better knowledge of how the user works. In contrast, the increased message handling in home health care (scenario 1) was not deliberately introduced, and its effect on the time and effort needed to process the messages was not acknowledged or understood until the system came into practical use.

#### Insight 2: HCI Design Principles—Independent of User, Task, and Context—Are Not Adopted

A user-centered process is always desired, but even without such a process there are *fundamental HCI design principles* that should be adopted. A long list, as shown in Figure 1, is a long list regardless of the specific user and context. Offering a list that is not searchable or does not adapt to the user’s most commonly chosen items is *inefficient* and can always be considered poor design. To increase usability, the list could have been either (1) sorted by intervention names instead of codes, (2) searchable by typing the first letters of the intervention name, or (3) sorted with the most commonly used interventions at the top of the list. The extensive clicking described in scenario 1 reveals a *complex information hierarchy* with many levels of information and interaction procedures that are not optimized for the task. *Inconsistency* in the system (in scenario 2), with different ways of presenting the signing information in one view compared with another (Figure 2, Figure 3), is also undesirable. Designing the user interface in this way is not in line with HCI design principles, where *efficiency*, *consistency*, and supportive *information structures* are examples of fundamental rules. Existing HCI design principles are based on information about human cognitive processes; by contravening these principles, interactions with the system occupy cognitive resources that could be used for better purposes [28-30,43].

#### Insight 3: Workarounds Become Permanent Solutions

In scenario 1, the nurse must fake a printing procedure to create a PDF that presents the patient’s health record in the desired chronological order. This solution works around the current default, which presents the information in reverse chronological order, not in line with how the nurse reads the information. It is not technically difficult to implement this correction, so one wonders why it was implemented incorrectly from the beginning. This could have happened because of a lack of understanding of the user’s tasks, revealing that a user-centered processes did not guide the system development. Since redesigning the software entails additional costs, a so-called workaround solution—creating a PDF—is offered. Likewise, the medical laboratory assistant in scenario 2 had created a workaround solution for handling the long list of lab tests that caused many incorrect orders. This solution involved a printed paper with a shorter version of the list, including only the available lab tests. These examples of workaround solutions are indicative of faulty system implementation and should lead to changes in the digital system. Instead, it is the users who must adapt, and the risk is that such workarounds can become a standard part of the interaction [3].

#### Insight 4: Different Domains of Ergonomics Interact in Creating the Digital Work Environment

Although HCI is traditionally associated mainly with the domain of cognitive ergonomics, we would like to emphasize that the development, implementation, and usage of a digital system entails interaction between several ergonomic domains: physical, cognitive, and organizational ergonomics [44]. This view is in line with what is called mesoergonomics, combining microergonomics, represented by physical and cognitive ergonomics at the individual level, and macroergonomics, represented by organizational aspects at the sociotechnical system level [45].

In the scenarios, we see examples of the users having to traverse long lists of information, interpret vague feedback, wait for the system to load, and be interrupted in tasks, all of which are related to cognitive ergonomics. Time-consuming or cumbersome interactions are frustrating and tiring to deal with and increase the cognitive load [43]. Delays of longer than one second in the home health care EHRs are a problem since time is a main determinant of cognitive load and mental effort [46].

Physical ergonomics is also part of the interaction. A design that involves small buttons, many clicks, and an inefficient layout, as in the two scenarios, can cause strain, mainly in the neck and shoulders [47,48]. The scenarios also present examples related to visual ergonomics, such as tiny user interface components, which may cause eye strain [49].

How tasks are performed in the EHR system is closely connected to, for example, how work is performed and how staff interact and communicate. We saw examples of this in scenario 1, in how communication was performed through messaging in home health care, and in scenario 2, when the physician at the primary care center had to allot extra time for administration in order to handle the new way of signing posts in the system. Hence, organizational aspects of ergonomics are also part of the digital work environment [5,45].

### Analysis of the Four Insights

What the four above insights share is that none of them is really novel: they all concern issues that have long been known, and in a sense solved, by HCI and ergonomics researchers. The insights were extracted from observations of real use situations involving modern EHRs and typical everyday work by health care professionals, meaning that the related issues are still very real for the typical users of such systems. Furthermore, several recent scientific publications support the insights noted in the scenarios, and we conclude that usability issues are still present, workarounds are still common, and there are no indications that these issues are decreasing in prevalence [3,5,22,24,50]. The systems, furthermore, introduce *new* tasks that must be performed in relation to the digital system [51], potentially making the work situation more complex rather than more efficient.

Concerning the first insight about introducing *inefficient workflows*, it would have been valuable if the practical implications of the new functionalities had been better understood before the implementation. This could partly be due to *lack of user studies and user involvement* in the design process [27]. Another factor could be the *lack of procurement requirements* regarding nonfunctional aspects, such as workflow, being formulated during system procurement [52]. This indicates a need for support when formulating requirements in the procurement process, to ensure both adequate user involvement and consideration of current workflows and work practice demands. Hence, requirements engineering and procurement strategies that ensure good usability and ergonomically sound systems clearly need more attention.

The second insight about violating *HCI design principles* differs in that the problem and, in principle, the solution are very clear. HCI design principles should be followed, and there are methods such as heuristic evaluation and cognitive walkthrough that can help designers and developers determine whether they have been implemented correctly [29,53]. Again, part of the issue is that these principles are insufficiently highlighted in the procurement process. Another aspect of the issue is that it can be hard for users to determine whether HCI design principles are violated in existing systems, so they are easy to miss when procuring new systems. While there are tools for developers and designers to evaluate this, they might not always be suitable for users and their organizations.

The third insight about *workarounds that become permanent solutions* has origins similar to those of the first and second insights—namely, their causes should be sought in the procurement process and in the evaluation of existing systems. Workarounds exist because the practical implications of new functionalities were not considered when the system was designed. Later, when the system is implemented in practice, tools are lacking for evaluating the system and pinpointing how the workarounds negatively affect practice. Tools and methods for evaluating digital systems that can be used in practice are important, as they *legitimize* the identification of issues with digital systems that affect health care practice.

The fourth and last insight concerns the interaction between *different domains of ergonomics*. While HCI theory is largely based on cognitive ergonomics, both physical and organizational ergonomics are involved when introducing new digital tools. As noted above, the EHR design used in scenario 2 actually forced the physician to *reorganize* her work to manage the signing task. Regarding physical ergonomics, using established HCI design principles such as Fitts’ law [54] to evaluate buttons and guide the layout and using visual ergonomics guidelines [55] on, for example, font size and color schemes, would be good starting points [56].

As the ongoing digitalization introduces new ways of working, including new hardware (eg, mobile documentation on tablets or wearable sensors) as part of the work equipment, interaction between different ergonomic domains can be expected to become more complex. A few relevant studies have already emerged. Johnson et al [57] identified a risk of musculoskeletal injuries when exchanging mouse and keyboard for a wearable sensor arm band. Xue [58] reviewed use areas and possible risks of wearables and also identified musculoskeletal strain as a possible risk.

## Paths Forward

### Overview

Based on the above discussion, we identify three paths toward a more sustainable digital work environment. The first path focuses on providing HCI theory–based *tools usable in health care practice to evaluate digital systems* as they are used in the field. The second path focuses on specifying *generic requirement formulations* related to usability, for use in the procurement process. The third path advocates introducing *digital ergonomics* as an embracing concept to emphasize all the ergonomic aspects involved in the digitalization process.

### Path 1: Tools for Evaluating Digital Systems in the Field

Studies exploring system usability are typically based on the users’ subjective grading of various usability statements related to the system (eg, [22,50]). This lets us know that users are sometimes frustrated when using digital systems. However, the specific reasons why a system is considered good or bad are not clearly identified: Is it because the design violates HCI design principles, because the workflow does not match the user’s task, because the system’s content layout does not fit the size and shape of the screen, or simply because the system is technically unstable? Furthermore, it is unreasonable to expect users in health care practice to identify HCI and ergonomic issues. In contrast, ergonomists, human factor specialists, and others responsible for the work environment should be able to do this with the right tools. They already perform risk assessment using ergonomic tools that, for example, measure lighting, musculoskeletal strain, and cognitive load, tools developed from a vast knowledge base in the related scientific subfield and adapted to be practically applicable. Some attempts are being made to support field assessment of the digital work environment [59,60], but we lack mature tools applicable *in practice by practitioners*. Revising existing principles and guidelines from HCI theory is required to transform these from theoretical, highly specialized methods into practically applicable tools for persons who are not HCI experts [61]. With such tools, it will be possible to evaluate an existing system and categorize and prioritize among the identified issues. The results of such an evaluation can then be used either to learn about the current digital work environment and make improvements based on this, or as a source of information when procuring new systems.

### Path 2: Generic Requirement Formulations

When procuring a new digital system, such as an EHR, requirements related to HCI and ergonomics are often either completely lacking or expressed in unverifiable terms [7]. Borg et al [62] showed that, in procuring digital tools, authorities primarily focus on functional requirements (what the system should do) rather than on quality demands (how well the functions are supported) such as usability. On the software development side, functional requirements are prioritized over quality-related requirements [63]. It has also been shown that requirements are lost during the software development process due to communication gaps [64]. There is apparently a need for support when it comes to formulating distinct and verifiable requirements for usability and ergonomics.

A lack of adequate *systems acquisition competence* is often identified as an explanation for bad digital systems being acquired [65]. It has even been suggested that health care professionals themselves should be more active in developing digital systems by, for example, taking responsibility for managing and designing the systems [66]. However, health care professionals are not, and should not need to be, specialists in software development, ergonomics, or design. Furthermore, it is unreasonable to expect all organizations acquiring digital systems—ranging from small home health care units and local primary care centers to large regional hospitals—to possess the extensive competence that is required to do this.

By creating guidelines for procurement that include user involvement, together with *generic requirement formulations* concerning usability and ergonomics, which can basically be taken “off the shelf,” it becomes easier to address those types of needs when specifying requirements. In this way, the risk of overlooking these aspects or losing track of them along the way decreases. Currently, no such generic requirement formulations exist; they need to be created from the current knowledge base regarding different aspects of ergonomics.

### Path 3: Digital Ergonomics—A Comprehensive Ergonomics Approach to Digitalization

As mentioned above, ergonomics has many aspects, and frameworks for combining these aspects have been proposed to allow for the simultaneous achievement of individual employee and organizational goals [45]. Digitalization has transformed work life and is itself affected by all levels of ergonomics. The examples from the studied scenarios indicate that ergonomic aspects are not fully considered in the digitalization process, resulting in poorly designed systems with suboptimized workflows causing unnecessary physical and mental strain. Increasing the awareness of ergonomic aspects in the digitalization process is necessary, and applying a holistic systems ergonomics perspective is desired to capture the complexity surrounding the digital work environment.

To meet this need, we propose the introduction of *digital ergonomics* as a concept used to emphasize the importance of adopting a comprehensive ergonomics approach, including relevant aspects from each subdomain of ergonomics, to develop sustainable digital work environments. We propose the following definition: *Digital ergonomics is the multidisciplinary science concerned with the application of theory, principles, data, and methods to the design of digital systems and the digital work environment, in order to optimize human well-being and overall system performance.*

This definition is based on the International Ergonomics Association’s [67] definition of ergonomics, adapted to narrow the focus on the implementation and usage of digital systems at work. Digital systems and their surroundings are arguably already part of ergonomics, as ergonomics as a discipline is rooted in sociotechnical values. However, since both HCI and ergonomic issues continue to emerge in health care practices and other work practices, an effort to direct attention to this area is needed. Uniting around the *digital ergonomics* concept would help users and developers focus on and clarify the required conditions for developing knowledge, tools, and methods for systematically addressing the work environment in relation to digitalization.

## Conclusion

Discussion of the digitalization of work is often general and insufficiently detailed to capture the full complexity of digitalization, which includes everything from effects on efficiency, safety, ethics, power relations, the work environment, and new ways of organizing work to designing the technology itself. Much research into digitalization emphasizes how health care is being transformed as a practice. As demonstrated in the above scenarios, many problems associated with digitalization only appear in actual use situations, where they also become issues for individual workers. Furthermore, many of these problems are completely unnecessary, since they are not novel and knowledge of how to avoid them has long existed.

In order to bridge this gap, initiatives are needed to more actively integrate ergonomics and HCI theory into practice. We suggest three paths forward so as not to overlook fundamental aspects of implementing and using digital systems in order to create a sustainable digital work environment: (1) development of *tools for evaluating the digital work environment in the field* capable of capturing details of the actual usage of digital systems, based on HCI theory, while being applicable in practice; (2) *generic requirement formulations* to ensure that demands related to all aspects of the digital work environment are considered in the procurement process and not lost sight of along the way; and (3) the introduction of the concept *digital ergonomics* in order to extract and merge aspects of all ergonomics domains that may influence the digital work environment. These three concrete suggestions would help bridge the gap between research-based knowledge and effects in practice in order to achieve sustainable digital work environments.

## Abbreviations

EHR: electronic health care record
HCI: human-computer interaction
